# Anterograde Amnesia during Electroconvulsive Therapy: A Prospective Pilot-Study in Patients with Major Depressive Disorder

**DOI:** 10.1371/journal.pone.0165392

**Published:** 2016-10-21

**Authors:** Elvira Boere, Astrid M. Kamperman, Arianne E. van 't Hoog, Walter W. van den Broek, Tom K. Birkenhäger

**Affiliations:** Department of Psychiatry, Erasmus Medical Center, Rotterdam, The Netherlands; University of Oxford, UNITED KINGDOM

## Abstract

Electroconvulsive therapy (ECT) is considered an effective treatment for major depression with melancholic features. However, neurocognitive side-effects such as anterograde amnesia still regularly occur. The present study aims to evaluate the severity and course of anterograde amnesia in severely depressed patients undergoing ECT. In a prospective naturalistic study, anterograde memory function was assessed among inpatients who underwent ECT (n = 11). Subjects met DSM-IV criteria for major depressive disorder. Recruitment took place between March 2010-March 2011 and March 2012-March 2013. Controls treated with antidepressants (n = 9) were matched for age, gender and depression severity. Primary outcome measure was immediate recall; secondary outcome measures were delayed recall, recognition, and visual association. Differences were tested using repeated measures ANOVA and paired t-tests. Correlations with hypothesized covariates were calculated. In patients with major depressive disorder, ECT had a significant effect on delayed memory function (p<0.01 with large effect sizes). Findings on immediate recall were less consistent. Four weeks after treatment discontinuation, these memory functions had recovered. Age was identified as a very important covariate. The main limitations of our study are its naturalistic design, possibly compromising internal validity, and its small sample size. However, if these findings can be reproduced in a more comprehensive study group, then the possible induction of anterograde amnesia is not a justifiable reason for clinicians to disregard ECT as a treatment option.

## Introduction

Electroconvulsive therapy (ECT) is considered an effective treatment for major depression with melancholic features (MDm), especially in psychotic depression [1]. ECT is also effective when previous pharmacotherapy has failed [2] and is relatively well tolerated [1]. However, despite multiple technical adjustments over the decades, neurocognitive side-effects still regularly occur. These may consist of decreased functioning in both anterograde and retrograde memory, as well as postictal disorientation and impairments in processing speed, verbal fluency, attention and executive functioning [3]. Although the underlying mechanisms remain to be elucidated, it is suggested that these side-effects may be temporary [4].

In a meta-analysis, reasonable evidence was found that cognitive side-effects associated with ECT are limited to the first three days after finishing ECT and that afterward, most improve beyond baseline [3]. However, primary studies on the particular subject of anterograde amnesia in relation to ECT are inconclusive concerning its severity and course [5–7].

An association has been reported between MDm and neurocognitive impairment, also in patients who did not receive ECT. In particular, explicit memory seems to be affected which may cause anterograde and retrograde amnesia, whereas implicit memory seems to be spared [8].

In this context, however, anterograde amnesia has not yet been extensively studied.

Our study therefore focuses on anterograde amnesia in severely depressed inpatients who are treated with ECT, focusing on both its severity and course. To meet our goals, we formulated the following research aims: 1) to compare the severity of anterograde amnesia during and after ECT with pharmacotherapy in severely depressed inpatients, and 2) to compare the course of anterograde amnesia during and after ECT with pharmacotherapy in severely depressed inpatients. We hypothesize that 1) anterograde amnesia will be of greater severity in ECT, compared with pharmacotherapy and that 2) compared to baseline, the severity of anterograde amnesia in ECT will increase compared with pharmacotherapy.

## Methodology

### Patient selection

#### Design

The course and extent of anterograde amnesia following ECT were studied using a naturalistic prospective design in patients with MDm. Patients treated with ECT were compared with a control group of depressed inpatients treated with antidepressants. This study was carried out in accordance with the latest version of the Declaration of Helsinki and approved by the Medical Ethics Committee of Erasmus Medical Center. All patients provided written informed consent after the study procedures had been fully explained.

#### Participants and setting

Recruitment took place between March 2010-March 2011 and March 2012-March 2013. Inpatients at Erasmus Medical Center diagnosed with MDm either with or without psychotic features, and for whom ECT was indicated, were considered eligible to participate: patients aged 18–90 years who consented to participate were included. During the second year (2012–2013) inpatients at Delta Psychiatric Hospital (Rotterdam, The Netherlands) meeting the same criteria were also considered eligible and were included after providing informed consent. The control group consisted of inpatients with MDm (with or without psychotic features) who were matched for age, gender and depression severity. Diagnosis was confirmed with the Schedule of Affective Disorder and Schizophrenia [9] and depression severity was assessed with the Hamilton Rating Scale for Depression [10].

#### Exclusion criteria

**Fig 1:**
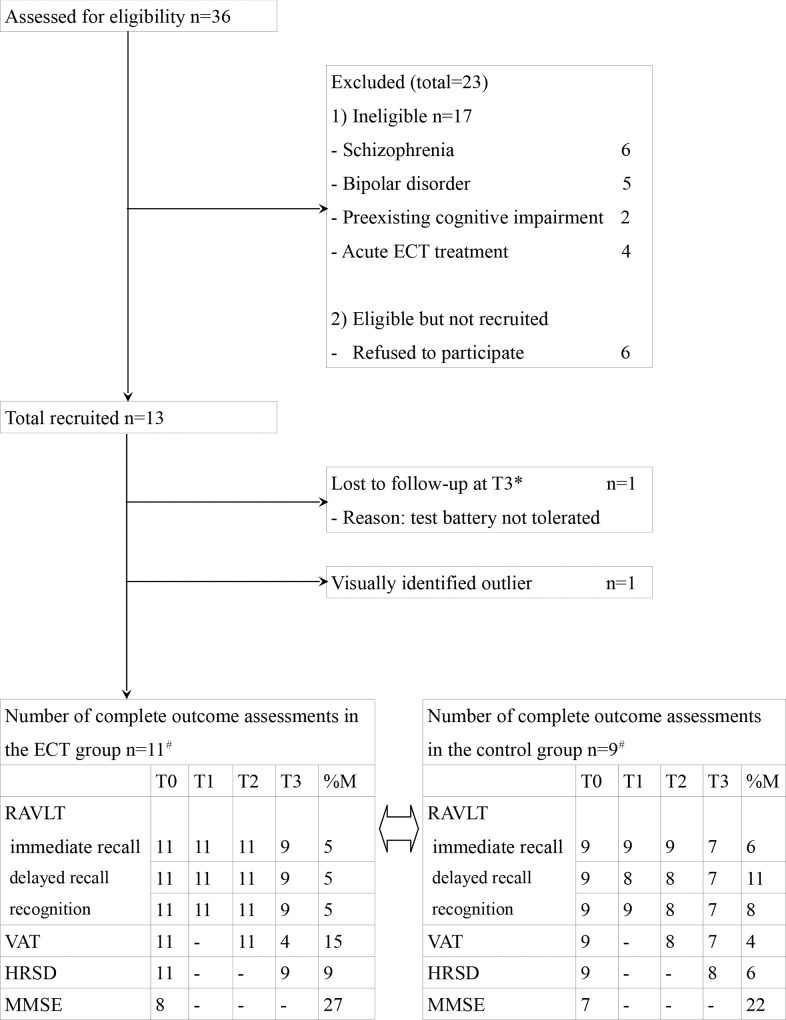
Flowchart of patient selection in the electroconvulsive therapy (ECT) group. %M = proportion of missing values over T0-T3; ^#^incomplete data were replaced by mean values; *assessments are defined as follows: T0 = baseline. In the ECT group, T1 = 2 weeks after start of ECT; T2 = 4 weeks after start of ECT; T3 = 4 weeks after discontinuation of ECT. In the control group, assessments are defined as follows: T1 = 2 weeks after reaching adequate blood level of antidepressants; T2 = 4 weeks thereafter; T3 = 8 weeks thereafter. RAVLT = Rey Auditory Verbal Learning Test; VAT = Visual Association Test; HRSD = Hamilton Rating Scale for Depression; MMSE = Mini Mental State Examination.

Patients admitted for acute ECT treatment were excluded as they could not be given sufficient time to consider their participation. Patients with pre-existing cognitive impairment, defined as a baseline score ≤ 24 on the Mini Mental State Evaluation (MMSE, range 0–30), were also excluded [11]. Other exclusion criteria were co-morbid alcohol or drug abuse, and a lifetime diagnosis of dementia or other cognitive disorder. The use of lithium was also considered as a possible exclusion criterion, because an adverse effect on cognitive functioning has been suggested [12]: however, as this is topic is still under debate, we decided to include these patients. Fig 1 summarizes the patient selection procedure.

### Interventions and clinical evaluation

Patients in the ECT group were withdrawn from all psychotropic medication at least 1 week before the first ECT treatment and maintained drug-free during the course of ECT. ECT was performed with the Thymatron System IV (Somatics Inc, Lake Bluff, Ill, USA); pulse width was set at 0.5 msec and energy used varied from 150 mC to 750 mC (mean dose of first ECT treatment was 75 mC; mean value throughout the course of ECT was 305 mC). The mean number of ECT treatments was 12.6 (median 14). Anesthesia was achieved with intravenously administered etomidate (0.2 mg/kg) and succinylcholine (1.0 mg/kg) for muscle relaxation. Bilateral ECT was administered twice a week. During ECT, physiological monitoring included pulse oximetry and blood pressure measurement, and an electrocardiogram. Seizure duration was determined using a 2-lead electroencephalograph and the cuff technique [13]. During the first ECT treatment, the seizure threshold was determined with stimulus titration. If the starting stimulus dose failed to elicit a seizure of at least 25-s duration measured with the cuff technique, the stimulus dose was increased according to the titration schedule (Table 1) and the patient was restimulated after 30 s. For the second treatment, the stimulus dosage was set at 1.5 times the defined seizure threshold. During the ECT course, stimulus dosage settings were adjusted upward to maintain a seizure duration of at least 25 s as measured with the cuff method. ECT was continued until patients were either asymptomatic (HRSD <8) or showed no further improvement (i.e. decrement on the HRSD) during 3 consecutive treatments.

**Table 1:** ECT dose titration schedule.

Dose level	Energy level (%)	Charge (mCoulomb)
1	5	25
2	10	50
3	20	101
4	40	202
5	60	302
6	80	403

At age < 50 the schedule is started at dose level 1, at age ≥ 50, the schedule begins with dose level 2.

Patients in the ECT group were tested at least 24 h after an ECT session. If patients in the control group used psychotropic medication at the time of inclusion, this medication was stopped at least 1 week before receiving a new course of treatment with antidepressants. Dosing was based on the following predefined blood levels: 200–300 ng/ml for imipramine, 50–150 ng/ml for nortryptyline and 0.60–0.80 mmol/L for lithiumcarbonate. Tranylcypromine was dosed at 70 mg/day. In all patients, depression severity was routinely assessed every week during treatment with the 17-item HRSD [10]: for the present study we collected data following the scheme presented by Fig 1.

### Outcome measures

#### Selection of tests

There is little evidence as to which tests adequately measure anterograde amnesia in depressed patients undergoing ECT. One study reported that verbal learning tasks are relatively sensitive to the effects of ECT [14]; these authors also found that immediate and delayed recall-subtests of the Rey Auditory Verbal Learning Test (RAVLT; see below) could adequately detect cognitive changes in patients who underwent ECT [15,16]. For the present study, this was an important consideration to choose the RAVLT as our main test. Because psychometric characteristics are slightly in favor of the immediate recall subtest [17], scores on this subtest are our primary outcome measure. Nonverbal memory tests are reported to have inconsistent sensitivity to ECT [14]. The Visual Association Test (VAT) adequately detects dementia at an early stage but (as far as we know) has not been tested in our specific subgroup of patients [18]. The VAT was used to validate findings on our primary outcome measure. With reassessments, because scores on memory tests may be influenced by a learning effect, alternating versions of the RAVLT and the VAT were used to reduce this effect to a minimum [17].

#### Primary outcome measure

Immediate recall, subtest of the RAVLT: translated Dutch version [16].

This test has high internal consistency (Cronbach's α > 0.80) and high test-retest reliability (*r* ranges from 0.61–0.86) [17]. The assessor sums up a list of 15 unrelated monosyllabic words repeated over five different trials: participants are asked to repeat these after each trial (scores range from 0–15, with higher scores indicating better anterograde memory function).

#### Secondary outcome measures

1. Delayed recall, subtest of the RAVLT: 20 min after the fifth trial (see **Primary outcome measure**). Delayed recall is tested by asking the participant to repeat the previously mentioned 15 words again (range 0–15).
2. Recognition, subtest of the RAVLT. Recognition is tested by summing 30 words, 15 of which were in the previous trials: score on recognition is defined as the total number of correctly identified words added to the total number of correct rejections (range 0–30). Test-retest reliability correlation coefficients for delayed recall and recognition subtests range from 0.51–0.72 [17].
3. Immediate recall by the VAT [18]: for this test the internal consistency (Cronbach's α > 0.80) and test-retest reliability (*r* = 0.72) are high. The test consists of six cue cards which depict one object, these cards are shown first. Then six association cards are shown, which depict a combination of the cue plus a target object. After this, the six cue cards are shown again; the participant is then asked for the (now missing) target object. Score ranges from 0–6, with a maximum score indicating intact anterograde memory function.

### Covariates

Because age [19], the presence of psychotic features [20] (confirmed by the SADS [9]), and severity of depressive symptoms [21] (measured by the HRSD [10]) may affect cognitive functioning, these were defined as covariates. With regard to depression severity, both baseline severity and percent change of mood compared to baseline during the study were tested as covariates.

### Statistical analysis

Differences between the ECT and control group at baseline were tested with t-tests for continuous variables (age, depression severity) and Chi^2^ or Fisher exact tests (FET) for categorical variables (psychotic features, gender). At each assessment, differences in cognitive functioning between assessments, and differences between the ECT and control group, were tested using (paired) t-tests. Subsequently, repeated measures ANOVAs were used to test whether the course of cognitive functioning differs over time between the two groups. Data were checked visually for non-normality using Q-Q plots. Missing data were replaced by the mean value. Correlations between the covariates and outcome measures were assessed by calculating Pearson’s *r*. Furthermore, the variable was entered as a covariate into the ANOVA analysis. Additionally, Cohen’s *f* for ANOVA and Cohen’s *d* for differences in means [22] were used to determine effect sizes: an *f* of 0.1 and a *d* of 0.2–0.3 is considered a small effect, an *f* of 0.25 and a *d* around 0.5 a medium effect, an *f* of 0.4 and a *d* of 0.8 and higher as a large effect.

### Power calculation

Very few studies quantified the impact of ECT on anterograde amnesia. In line with a more recent study by Bodnar et al. [23], we decided that a 5 point difference between ECT and control patients on mean scores on the RAVLT over the full course of the study could be interpreted as a clinically significant increase of anterograde amnesia. This equals to a medium sized treatment*measurement interaction-effect (*f* = 0.33). To detect a medium-sized effect at 5%-significance level and with a power of 80% (assuming a correlation among the 4 measurements of r = 0.25), we needed a sample size of 20 patients. All calculations were performed with IBM SPSS Statistics 20.

### Follow-up after recruitment

Initially, 13 patients were recruited in the ECT group and 11 in the control group.

In the ECT group, 1 patient dropped-out and 1 was visually identified as an outlier, leaving11 patients available for analysis. In the control group, 1 patient dropped-out and 1 was visually identified as an outlier, leaving 9 patients available for analysis. One additional patient from the control group was excluded from the analysis for the VAT due to dropout after baseline measurement: therefore, 8 control patients entered the analysis.

The number of subjects with previous pharmacotherapy failure was 10 in our ECT group and 6 in our control group. One patient in our ECT group was diagnosed with a comorbid obsessive compulsive disorder, for which clomipramine was continued during the study: all other included patients were diagnosed with MDm only.

Two patients in the ECT group were recruited at the Delta Psychiatric Hospital and all others (in both groups) were recruited at the psychiatry ward of Erasmus Medical Center.

Regarding covariates, the number of included patients with psychotic features was too small to determine correlation or to perform further analyses. Although we considered making an additional assessment at 6 months after discontinuation of ECT, because dropout rates at that time were very high we did not include this assessment in the present analysis. Fig 1 presents an overview of the flow of participants through the study.

## Results

### Group characteristics

The analyses included 11 patients undergoing ECT and 9 patients treated with antidepressants. Matching for age, gender and depression severity was successful (Table 2).

**Table 2:** Characteristics of the two study groups.

	ECT (n = 11)	Control (n = 9)	
*Demographics*			
Male:female	5:6	3:6	FET: p = 0.67
Mean age in years (SD)	59.55 (15.3)	56.11 (12.1)	T(18) = 0.55; p = 0.59
*Primary diagnosis at baseline*			
MDm without psychotic symptoms	8	8	FET: p = 0.59
MDm with psychotic symptoms	3	1	
*Depression severity and global cognitive functioning*			
HRSD (SD) baseline	23.64 (5.3)	25.00 (3.1)	T(18) = 0.68; p = 0.50
HRSD (SD) at T3^a^	12.30 (5.8)	16.88 (6.4)	T(18) = 1.67; p = 0.11
%change HRSD (SD)	0.46 (0.3)	0.30 (0.3)	T(18) = 1.07; p = 0.30
MMSE (SD) baseline	27.38 (2.5)	28.00 (2.9)	T(13) = 0.45; p = 0.66
*Treatment*			
ECT–right unilateral	1^b^	-	
ECT–bilateral	10	-	
imipramine	-	4	
nortriptyline	-	2	
imipramine and lithium	-	2	
tranylcypromine	-	1	
clomipramine	1^b^	0	
mirtazapine and lithium	1^b^	0	

ECT = electroconvulsive therapy; MDm = major depression with melancholic features; HRSD = Hamilton Rating Scale for Depression; MMSE = Mini Mental State Examination; FET = Fisher’s exact test.

^a^ T3 in the ECT group is defined as 4 weeks after discontinuation of ECT and in the control group as 8 weeks after reaching adequate blood level of antidepressants.

^b^violation of protocol.

### Anterograde amnesia

#### Covariates

Age. In the ECT group, age at baseline was strongly correlated with immediate recall (r = 0.84; p<0.01) and in the control group these variables were moderately correlated (r = 0.35; p = 0.36).

Depression severity. In the ECT group, baseline HRSD scores were positively correlated with immediate recall (r = 0.60; p = 0.05), whereas in the control group baseline HRSD scores were negatively correlated with immediate recall (r = -0.44; p = 0.23). This was not consistent with our expectations and is discussed below.

Change in depression severity. In both groups, percent change in depression severity was negatively correlated with scores on immediate recall. In the ECT group we found a moderate correlation (r = -0.28; p = 0.41) and in the control group a weak correlation (r = -0.12; p = 0.76).

#### Primary outcome measure: immediate recall

**Fig 2:**
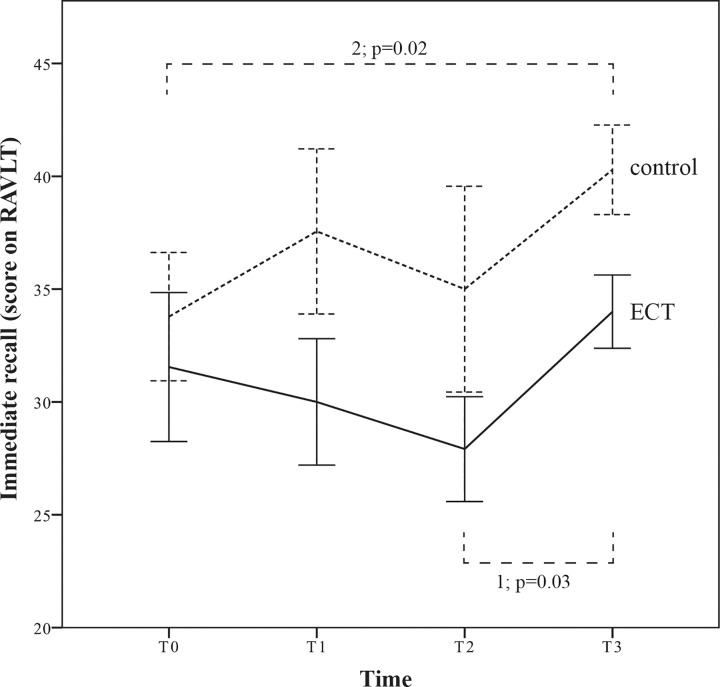
Results for repeated measures ANOVA on immediate recall (with +/- 1 SE). Repeated measures ANOVA showed no significant differences. However, the within-group effect (using paired t-tests) was significant between T2 and T3 in the ECT group (1; p = 0.03) and between T0 and T3 in the control group (2; p = 0.02). T0 = baseline. In the ECT group, T1 = 2 weeks after start of ECT; T2 = 4 weeks after start of ECT; T3 = 4 weeks after discontinuation of ECT. In the control group, assessments are defined as follows: T1 = 2 weeks after reaching adequate blood level of antidepressants; T2 = 4 weeks thereafter; T3 = 8 weeks thereafter.

We found a significant effect over time (F(3,54) = 3.960; p = 0.013), but not between both groups (F = 2.568; p = 0.126). The data showed no significant time*treatment interaction effect on immediate recall (F(3) = 0.976; p = 0.411; *f* = 0.23) (Fig 2).

Adjusting for age did not result in a significant time*treatment interaction effect (F(3) = 1.34; p = 0.27; *f* = 0.28), nor did adjustment for depression severity at baseline (F(3) = 1.00; p = 0.40; *f* = 0.24) or adjustment for mood change (F(3) = 0.83; p = 0.49; *f* = 0.22). However, adjusting for age considerably lowered the p-value and slightly increased *f*, suggesting that age may be a relevant factor in this study.

In the ECT group, immediate recall decreased at trend level statistical significance between T0 and T2 (T(10) = 2.00; p = 0.07; *d* = 0.38) and increased significantly between T2 and T3 (T(10) = -2.54; p = 0.03; *d* = 0.92). In the control group, immediate recall increased significantly between T0 and T3 (T(8) = -2.86; p = 0.02; *d* = 0.89). Compared to the control group, the ECT group scored lower at T3. Results are summarized in Table 3.

**Table 3:** Mean values (SD) of all outcome measures, between-group comparisons, and effect sizes at each time of assessment.

	ECT	Control		*d*
Immediate recall (n = 11; n = 9)				
T0	31.55 (11.0)	33.78 (8.5)	T(18) = -0.50; p = 0.62	-0.23
T1	30.00 (9.3)	37.56 (11.0)	T(18) = -1.67; p = 0.11	-0.74
T2	27.91 (7.7)	35.00 (13.7)	T(18) = -1.46; p = 0.16	-0.64
T3	34.00 (5.4)	40.29 (6.0)	T(18) = -2.48; **p = 0.02**	-1.11
Delayed recall (n = 11; n = 9)				
T0	6.5 (3.2)	6.3 (2.8)	T(18) = 0.16; p = 0.88	0.07
T1	5.3 (2.6)	7.9 (3.4)	T(18) = -1.98; p = 0.06	-0.86
T2	3.5 (2.4)	7.8 (3.8)	T(18) = -3.16; **p = 0.01**	-1.35
T3	6.5 (2.2)	7.7 (2.5)	T(18) = -1.15; p = 0.27	-0.51
Recognition (n = 11; n = 9)				
T0	27.3 (3.5)	26.3 (3.7)	T(18) = 0.58; p = 0.57	0.28
T1	24.7 (4.4)	26.4 (4.2)	T(18) = -0.89; p = 0.39	-0.40
T2	26.5 (3.1)	26.4 (4.7)	T(18) = 0.09; p = 0.93	0.03
T3	26.6 (3.7)	26.3 (3.3)	T(18) = 0.19; p = 0.85	0.09
VAT (n = 11; n = 8)				
T0	5.1 (1.1)	5.5 (1.1)	T(17) = -0.79; p = 0.44	-0.36
T2	5.2 (1.4)	5.0 (1.4)	T(17) = 0.28; p = 0.78	0.14
T3	5.5 (0.6)	5.6 (0.7)	T(17) = -0.35; p = 0.73	-0.15

**Bold** indicates significant value. T0 = baseline. In the ECT group, T1 = 2 weeks after start of ECT; T2 = 4 weeks after start of ECT; T3 = 4 weeks after discontinuation of ECT. In the control group, assessments are defined as follows: T1 = 2 weeks after reaching adequate blood level of antidepressants; T2 = 4 weeks thereafter; T3 = 8 weeks thereafter. Results are uncorrected for multiple comparisons.

#### Secondary outcome measures

Results for all secondary outcome measures are summarized in Table 3.

Delayed recall. We didn't find a significant effect over time (F(3,54) = 1.978; p = 0.128), nor between both groups (F = 3.552; p = 0.076). However, the time*treatment interaction effect on delayed recall was significant (F(3) = 5.43; p<0.01; *f* = 0.55), which can interpreted as a different course of delayed recall function in patients in the ECT group, compared to its course in control patients. Adjustment for age resulted in a very slightly lowered p-value and a slightly increased *f* (F(3) = 6.28; p<0.01; *f* = 0.61), and so did adjustment for mood change (F(3) = 6.08; p<0.01; *f* = 0.60). Adjustment for baseline depression severity resulted in a similar p-value and a very slightly increased *f* (F(3) = 5.48; p<0.01; *f* = 0.57).

In the ECT group, scores at T2 were lower compared to baseline (T(10) = 4.30; p<0.01; *d* = 1.06) and scores at T3 were higher compared to those at T2 (T(10) = -4.45; p<0.01; *d* = 1.30). Compared to the control group, the ECT group had lower scores at T2.

On the recognition and visual association tasks, no significant effect was found between assessments in the ECT group (*d* ranged from 0.03–0.64 and from 0.07–0.39, respectively) or between the ECT and control group (*d* ranged from 0.04–0.40 and from 0.13–0.37, respectively), and there was no time*treatment interaction effect (*f* = 0.01 and 0.06, respectively).

## Discussion

### Anterograde amnesia

The primary finding of our study is that the course of delayed recall function in severely depressed inpatients undergoing ECT is significantly impaired, compared to patients who are treated with antidepressants. Delayed recall function in the ECT group recovered within four weeks after treatment discontinuation. These results are consistent with an uncontrolled study [15], in which significant effects of ECT were found on immediate (n = 28) and delayed (n = 26) recall between the 3rd and 4th treatment session.

In immediate recall, we found a decrease of function during ECT, however, statistically this finding did not reach significance. Also, we found that immediate recall function improved within four weeks of treatment discontinuation. Our findings support the hypothesis that anterograde amnesia may be a temporary effect of ECT.

From baseline onward, immediate recall function was impaired in the ECT group compared to the control group, reaching significance (with a large effect size) four weeks after discontinuation of ECT. Although further recovery of immediate recall function could be expected, the study was terminated at this point due to a high dropout before the (planned) last assessment. In the control group, immediate recall improved significantly compared to baseline, which might be attributed to successful treatment of depression as well as to their treatment: these patients did not receive ECT so they were not exposed to the, in our article discussed, risk of cognitive deterioration caused by ECT.

In Fig 2, showing the within-group effects, a more gradual increase of immediate recall was expected in the control group. However, mean immediate recall at four weeks after reaching adequate blood level of antidepressants was lower (albeit not significantly) than at two weeks thereafter. Analysis of these data shows that this decrease might be due to the very low score of one patient only: after removal of this patient from the sample, the mean value at this assessment returned to a normal range.

In our study, both the RAVLT sub-test for recognition and the VAT failed to detect cognitive impairment. In the vast majority of assessments, scores on the VAT were either 5 or 6; as a score of 6 indicates a maximum-score on the VAT, this finding may be indicative of a ceiling effect of this test.

### Confounding factors

Higher age was strongly correlated with a decreased immediate recall function at baseline. This is consistent with an epidemiological cohort study reporting an association between depressive symptoms, and poor cognitive functioning and cognitive decline, in particular with advancing age [24]. Also, in the absence of depression, age has previously been found to have a significant effect on performance on the RAVLT [25]. Furthermore, we found that at lower age ECT may have a greater impact on immediate recall function. However, we need to consider the possibility of a floor effect of our test. Most patients in the control group used tricyclic antidepressants, which may induce cognitive impairment due to their anticholinergic effects [26] and, thereby, decrease differences in cognitive functioning between the two groups. However, the presumed cognitive effects of tricyclic antidepressants need further investigation. Percent mood change during the study was weakly, negatively correlated to improvement of immediate recall function. However, we also found that adjustment for this covariate resulted in a slight increase in the interaction effect on delayed recall function. Also, in the control group in our study, greater depression severity at baseline was positively correlated with a better immediate recall function. Although this seems counter-intuitive, depression severity may be unrelated to scores on the immediate and delayed recall subtests of the RAVLT [27]. Therefore, our finding might be a misleading result.

### Limitations

Methodologically, the main limitations of our study are its naturalistic design, possibly compromising internal validity, and its small sample size. Furthermore, replacing missing data by means may result in decreased variability of the data, leading to false-positive results. Some patients choosing not to participate based their decision on the notion that they considered themselves incapable/unworthy of participating. Because this might be explained by the severity of their depression, the most severely depressed patients may be underrepresented in the present study. In the ECT group, the protocol was violated in 3 (of 11) patients, which may have affected the outcome data. Although we aimed to match depressed patients receiving ECT with similar patients treated with antidepressants, these samples may have some inherent differences. Patients were matched for age, gender, and depression severity as measured with the baseline HRSD score. Nevertheless, patients receiving ECT are likely to differ from patients treated with antidepressants, i.e. they are either very severely depressed, or there is an acute indication for ECT (these patients were excluded from participation), or they did not respond to several adequate trials of antidepressants. Therefore, patients receiving ECT after failure of pharmacotherapy probably had a longer index episode than patients treated with antidepressants.

The researchers who collected data were not blinded for the treatment of study-subjects. In a systematic review comparing randomized clinical trials with both blinded and non-blinded assessors, the latter were found to have a higher risk of observer bias with subjective measurement scale outcomes [28]. In our study however, we adapted measurement tools with high test-retest reliability, in an attempt to reduce potential observer bias to a minimum. Also, an observer bias would hypothetically lead to overestimation of cognitive defects during assessments: since we only found small effects, the true effects of ECT on anterograde memory function may even be smaller than we described.

Although it would have been unethical to blind our subjects for their treatment, one may argue, that also study subjects may influence their scores on cognitive functioning tasks based on their expectancies. Studies on this particular matter are scarce, however, interestingly, a recent study found no effects of study subjects' expectation on objective cognitive measures [29]. Also, inherent to clinical research, a Hawthorne effect, in which study subjects tend to change their behavior when they enter a study, cannot be excluded. This effect may influence external validity of clinical studies and has been studied to a very small extent. However, identifying and quantifying this potential effect has proven to be very difficult [30].

Our studied patients received bilateral ECT, which has been associated with increased efficacy in the treatment of MDm compared to right unilateral (RUL) ECT [1]. Bilateral ECT has also been found to induce greater cognitive side effect than RUL ECT. However, in recent studies, higher dosages in RUL ECT have been proposed, which may decrease differences in cognitive side-effects between bilateral and high-dose RUL ECT. A recently published study found no difference in efficacy between bilateral and high-dose RUL ECT, whereas the latter induced less cognitive side-effects [31].

### Future directions

Future research on anterograde amnesia would benefit from including more study subjects and providing a longer follow-up, preferably up to 6 months after treatment discontinuation. The risk of relapse in this follow-up period however could pose an extra difficulty for the researchers in interpreting the results. Mood change as a covariate may need further exploration. Another focus for future research, although not discussed in our paper more extensively, may lie in the use of neuroprotective agents: galantamine may prevent anterograde amnesia in depressed inpatients who undergo ECT [32].

### Conclusion

In the present study, ECT had a significant effect on delayed memory function in patients with major depression with melancholic features. Effect sizes were large, suggesting that this effect may relatively easily be detected in clinical practice. Findings related to immediate recall function were less consistent. Four weeks after treatment discontinuation, these functions had recovered. Recognition and visual association were not affected, and age was identified as an important covariate. Overall, the clinical implications of anterograde amnesia caused by ECT may be small. If our findings can be reproduced in a more comprehensive study, then the possible induction of anterograde amnesia will not be a justifiable reason for clinicians to disregard ECT as a treatment option in these patients.
